# Characterization of the *Caenorhabditis elegans* HIM-6/BLM Helicase: Unwinding Recombination Intermediates

**DOI:** 10.1371/journal.pone.0102402

**Published:** 2014-07-18

**Authors:** Hana Jung, Jin A Lee, Seoyoon Choi, Hyunwoo Lee, Byungchan Ahn

**Affiliations:** Department of Life Sciences, University of Ulsan, Ulsan, Republic of Korea; National Cancer Institute, United States of America

## Abstract

Mutations in three human RecQ genes are implicated in heritable human syndromes. Mutations in *BLM*, a RecQ gene, cause Bloom syndrome (BS), which is characterized by short stature, cancer predisposition, and sensitivity to sunlight. BLM is a RecQ DNA helicase that, with interacting proteins, is able to dissolve various DNA structures including double Holliday junctions. A BLM ortholog, *him-6*, has been identified in *Caenorhabditis elegans*, but little is known about its enzymatic activities or its *in vivo* roles. By purifying recombinant HIM-6 and performing biochemical assays, we determined that the HIM-6 has DNA-dependent ATPase activity HIM-6 and helicase activity that proceeds in the 3'-5' direction and needs at least five 3' overhanging nucleotides. HIM-6 is also able to unwind DNA structures including D-loops and Holliday junctions. Worms with *him-6* mutations were defective in recovering the cell cycle arrest after HU treatment. These activities strongly support *in vivo* roles for HIM-6 in processing recombination intermediates.

## Introduction

The RecQ family of proteins belongs to superfamily 2 of DNA helicases, which was named after the *Escherichia coli* ortholog, RecQ [1]. Most prokaryotes have a single RecQ homologue, whereas eukaryotic organisms often possess more than one RecQ family member (reviewed in [2]. The RecQ helicases are implicated in maintaining genome stability across various species.

In *Caenorhabditis elegans*, there are 4 RecQ helicase members including HIM-6, WRN-1, RECQ1, and RECQ5 [2], [3]. The *C. elegans him-6* gene encodes a 988 amino acid BLM helicase ortholog. Worms with *him-6* mutations are radiation sensitive and exhibit genomic instability and decreased frequency of meiotic recombination. In addition, mitotically proliferating germ cells of *him-6* mutants show increased frequency of double-strand breaks (DSBs) [4]–[6].

Bloom syndrome (BS) is an autosomal recessive human genetic disorder caused by mutation of the Bloom syndrome gene (*BLM*). Cells from BS patients exhibit increased chromosomal abnormalities, frequency of sister-chromatid exchange (SCE), and recombination events. The BLM protein has 3'-5' helicase activity that unwinds forked DNA duplexes, a synthetic X-structure that models a Holliday junction (HJ), and G-quadruplex DNA. In addition, BLM dissolves double Holliday junctions (dHJs) with topoisomerase III alpha [7]–[10] and can regress a stalled or collapsed replication fork [11].

The *Drosophila melanogaster* ortholog of BLM, DmBLM, is encoded by the *mus309* gene. Mutations in the *mus309* gene cause hypersensitivity to DNA-damaging agents, female sterility, and defects in DSB repair [12], [13]. Purified DmBLM has 3'-5' DNA helicase activity, unwinds forked duplex DNAs, and has strand annealing activity [14]. In addition, DmBLM can resolve a mobile double HJ with topoisomerase III alpha [15].

The budding yeast *Saccharomyces cerevisiae* has a BLM homolog, Sgs1. *S. cerevisiae sgs1* mutants that are sensitive to genotoxic agents show increased chromosome missegregation and elevated mitotic recombination, suggesting that Sgs1 plays a key role in regulating mitotic recombination [16], [17]. Purified Sgs1 has 3'-5' DNA helicase activity and unwinds a wide variety of duplex DNAs, including blunt-ended duplex DNA and HJs [18], [19]. Recent studies have reported a second RecQ helicase, Hrq1, which is an ortholog of human RECQL4 [20], [21].

*C. elegans him-6* mutants display heightened sensitivity to ionizing radiation (IR) and a mutator phenotype similar to human BLM^−/−^ cells [5]. In addition, *him-6* mutants are predominantly male and have reduced meiotic recombination [6]. These genetic observations suggest that the HIM-6 protein may play a role in DNA repair and/or recombination. However, it is not yet known how the activity of HIM-6 is related to these phenotypes, and the biochemical activities of HIM-6 have not been investigated.

In this study, we investigated the biochemical activities of HIM-6. We expressed and purified recombinant HIM-6 protein and performed helicase and ATPase assays to determine biochemical functions. To connect the biochemical activities to phenotypes, we performed mitotic germ cell proliferation arrest assays. Our results will help uncover the roles of HIM-6 in *C. elegans*.

## Materials and Methods

### Protein, oligonucleotides, DNA substrates, and antibodies used in this work

PAGE-purified oligonucleotides modeling DNA helicase substrates were synthesized by IDT (Integrated DNA Technology, USA) and are listed in Table S1 in File S1. For each substrate, a single oligonucleotide was labeled at the 5'-end with [γ-^32^P] ATP (IZOTOP, Hungary). Oligonucleotides were incubated with T4 polynucleotide kinase (New England Biolabs, USA) for 1 hour at 37°C, followed by heat-inactivation for 10 minutes at 95°C. The labeled oligonucleotides were purified using G-25 Sepharose spin columns and annealed to unlabeled complementary strands (Figures S1A and S1B in File S1) at a molar ratio of 1∶3 by incubation at 90°C for 5 minutes followed by slow cooling to room temperature.

The HIM-6 antibody was prepared by antipeptide antibody production. Two polypeptides derived from amino acid residues 891–903 and 948–965 were selected and the polyclonal antipeptide antibody was produced from rabbits (Ab frontiers, Korea). A monclononal anti-6× His antibody was purchased from Sigma-Aldrich (USA).

### Cloning of *C. elegans* him-6 cDNA

An EST clone (yk287e5, NGI, Japan) that contained amino acid residues 21–988 of the *C. elegans him-*6 gene was used for polymerase chain reaction (PCR). PCR products were amplified by Pfu DNA polymerase (Strategene, USA) using forward and reverse primers, which were designed to clone into the pENTR/D/TOPO plasmid (Invitrogen, USA). The sequence and orientation of the *him-6* insert in the plasmid were confirmed by DNA sequencing (Bioneer, Korea). The cloned *him-*6 in pENTR/D/TOPO was transferred to the *E. coli* expression vector pDEST17 for HIS_6_-tagged protein purification using the Gateway LR Clonase II (Invitrogen, USA) and confirmed by DNA sequencing. The pDEST17 containing *him-6* was transformed into *E. coli* BL21AI for protein expression.

### Protein purification

The recombinant HIS_6_-tagged HIM-6 fusion protein was expressed in *E. coli* BL21AI. *E. coli* cells were grown at 37°C in 1 liter of Luria-Bertani media containing 100 µg/ml ampicillin to an OD_600_ of 0.4. L-arabinose (Sigma-Aldrich, USA) was added to the culture to a final concentration of 0.2% (w/v) to induce protein production, and the cells were grown for an additional 4 hours at 22°C. Cultured cells were harvested by centrifugation, suspended in 20 ml lysis buffer (50 mM Tris-HCl, pH 8.0, 500 mM NaCl, 0.5 mM EDTA, 5 mM β-mercaptoethanol, 10% glycerol, 1 mM PMSF, 1 protease inhibitor cocktail tablet (Roche, Mannheim, Germany), 1 mM imidazole) and lysed by sonication (duty cycle 20/output 2, 10 bursts at 10 second intervals, Branson). Lysates were clarified by centrifugation at 10,000×*g* for 30 minutes at 4°C. The cleared lysates (10 ml) were mixed with 2 ml of 50% Ni^2+^-NTA-agarose (Invitrogen, USA) and incubated on a rotary shaker at 4°C for 1.5 hours.

The lysate and Ni-NTA mixture was loaded into a small chromatography column (Takara, Japan). The column was washed with 10 column volumes of wash buffer 1 (lysis buffer plus 10 mM imidazole) and 10 column volumes of wash buffer 2 (lysis buffer plus 25 mM imidazole) to remove nonspecifically bound protein. HIM-6 was eluted with 5 ml of elution buffer (lysis buffer plus 300 mM imidazole). Peak fractions containing HIM-6 were detected using SDS-PAGE. Protein concentrations were determined using a Bio-Rad assay with BSA as the standard. The purified HIM-6 protein was verified using a MALDI TOF-TOF 4700 proteomics analyzer (Postech Biotech Center, Korea).

### Helicase Assay

Proteins and radiolabeled DNA substrates (10 fmol of labeled oligomers) were mixed in 10 µl of helicase reaction buffer (50 mM HEPES, pH 7.5, 20 mM KCl, 2 mM MgCl_2_, 2 mM ATP, 2 mM dithiothreitol, 0.1 mg/ml BSA). Reactions were incubated at 37°C for 15 minutes and terminated by the addition of 3× stop dye (0.05 M EDTA, 40% glycerol, 1% SDS, 0.05% bromophenol blue, 0.05% xylene cyanol FF dye). Helicase reaction products were analyzed by polyacrylamide gel electrophoresis (PAGE) using non-denaturing 10% polyacrylamide gels (19∶1 acrylamide:bisacrylamide, Bio-Rad labs, USA). Radiolabeled DNA species in polyacrylamide gels were visualized on Hyperfilm (Amersham, GE health, USA) and quantitated using Scion Image software (Frederick, MD, USA).

The percentage of DNA unwinding was calculated as the amount of released intact oligonucleotide (product) divided by the total amount of DNA substrate used in a helicase reaction. The values of the product and the substrate were corrected by subtracting the background values obtained from no enzyme and heat-denatured substrate controls, respectively. The helicase data represent the mean of at least 3 independent experiments ± standard deviation (SD).

### ATPase assay

The ATPase assay reaction mixtures (10 µl) contained 50 mM Hepes-KOH (pH 7.5), 20 mM KCl, 1 mM DTT, 2 mM MgCl_2_, BSA (0.1 mg/ml), DNA (250 ng of ssDNA or 250 ng of supercoiled duplex DNA), and HIM-6 or HIM-6 (K275A). Reactions were initiated by the addition of ATP to a final concentration of 2 mM and incubated at 37°C for the indicated times. Reactions were terminated by adding 0.1 M EDTA (pH 8.0) to a final concentration of 0.05 M and 100 µl of QuantiChrome reagent (Bioassay systems, USA). The terminated reaction mixtures (100 µl) were pipetted in duplicate into wells of a clear-bottom 96-well plate. After 5 minutes, the plates were scanned at 620 nm in a microplate ELISA reader (Wallac, Turku, Finland). The moles of Pi were determined from a calibration curve made from solutions containing known Pi concentrations (KH_2_PO_4_).

### Mitotic Germ Cell Proliferation Arrest

To observe nuclear morphology, worms were grown at 20°C to larval stage L4. L4 stage worms were transferred to a new nematode growth medium (NGM) plate containing 20 mM hydroxyurea (HU) and treated for 24 hours. Gonads were dissected at the indicated times after HU treatment and 4,6-diamidino-2-phenylindole (DAPI, 1 µg/ml) staining was performed. The stained gonads were observed with a Carl Zeiss Axioskop fluorescence microscope. A deletion allele created by the *C. elegans* Knockout Consortium, *ok412* and the deletion found in *him-6*(*ok412*) strain obtained from the *Caenorhabditis* Genetic Center (CGC, University of Minnesota, USA).

### Immunostaining

Worms were dissected in egg buffer supplemented with 0.4 mM levamisol (Sigma-Aldrich, USA). Germlines were transferred on a poly lysine coated slides then fixed in 2% paraformaldehyde for 5 min at room temperature followed by freeze cracking on a metal block submersed in liquid nitrogen. Post-fixation was done in methanol at −20°C. Blocking was performed by incubating the samples with Image-iT FX signal enhancer (Invitrogen, USA) for 20 min, followed by 15 min of incubation in PBSTB (PBS +0.1% Tween 20 (PBST) +0.5% BSA). Primary antibodies were diluted in PBSTB and allowed to bind at 4°C overnight in a humid chamber. Samples were washed three times for 10 min in PBST. Binding of secondary antibodies was performed for 2 h at room temperature with antibodies diluted in PBSTB supplemented with 1 µg/ml DAPI. After washing three times for 10 min in PBST, the samples were mounted in VECTASHIELD mounting media (Vector labs, USA). Pictures were taken on a Carl Zeiss Axioskop fluorescence microscope.

## Results

### Purification of HIM-6

**Figure 1:**
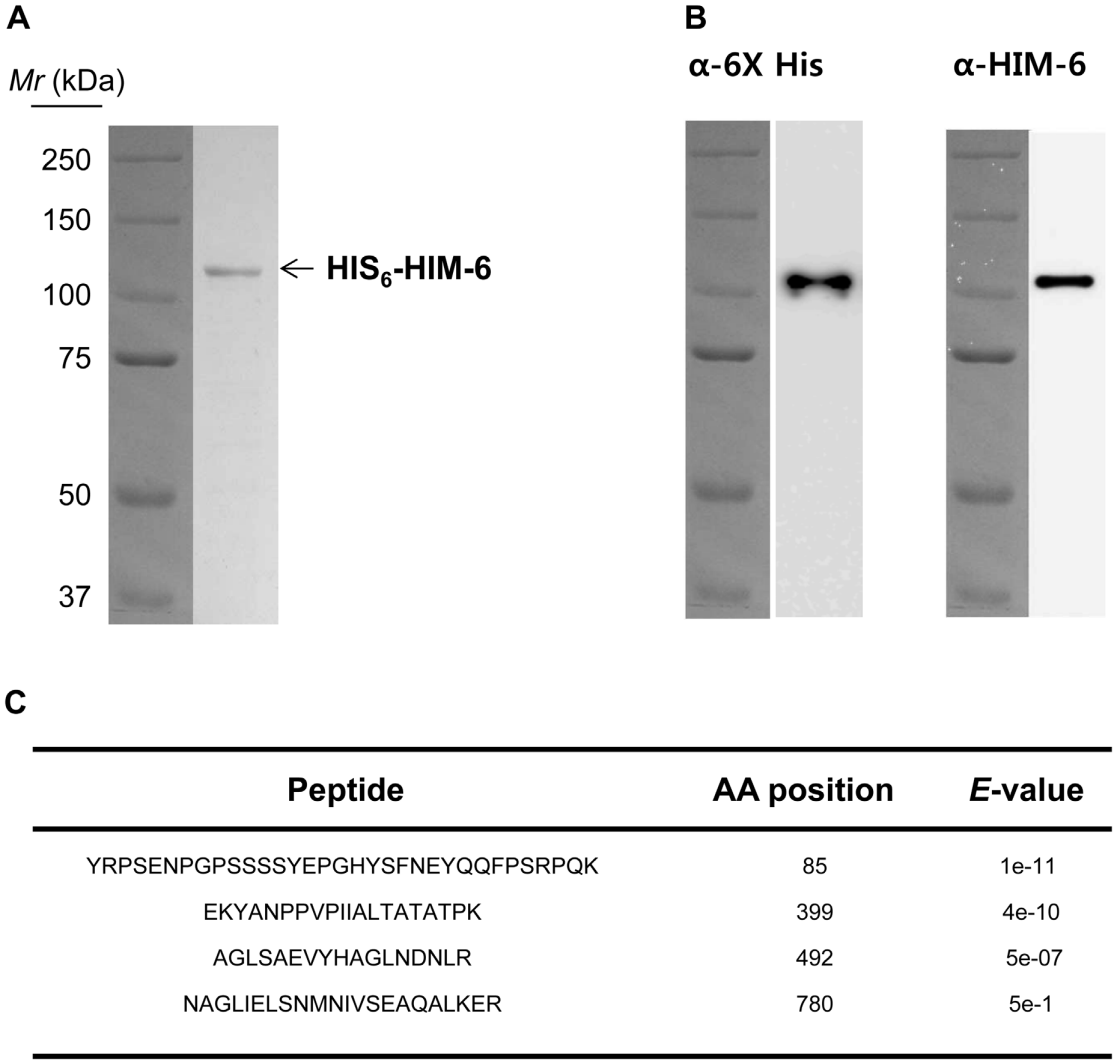
Purification of Recombinant HIM-6 protein. (A) SDS–PAGE analysis of purified HIM-6. Lane 1, relative molecular mass markers; lane 2, 1 µg purified HIM-6. (B) Western blot with anti-6× His and anti-HIM-6 antibodies. (C) MALDI-TOF analyses of 4 fragments of purified HIM-6. The starting amino acid (AA) positions of the fragments are numbered as indicated, and their corresponding *E* values are listed.

The *C. elegans him-6* EST clone containing amino acid residues 21-988 was fused with an N-terminal HIS_6_ tag, expressed in *E. coli*, and purified using Ni^2+^-NTA-agarose resin to approximately 95% homogeneity (Figure 1A). The recombinant protein had a theoretical molecular mass of 110 kDa and migrated at ∼110 kDa on a 7% polyacrylamide gel. Western blot analyses of the purified HIM-6 using a polyclonal anti-HIM-6 antibody and a monclononal anti-6× His antibody revealed a single band at ∼110 kDa (Figure 1B). MALDI-TOF analysis confirmed that the purified protein contained the amino acids encoded by the *him-6* gene (Figure 1C).

### HIM-6 is a 3'-5' DNA helicase

**Figure 2:**
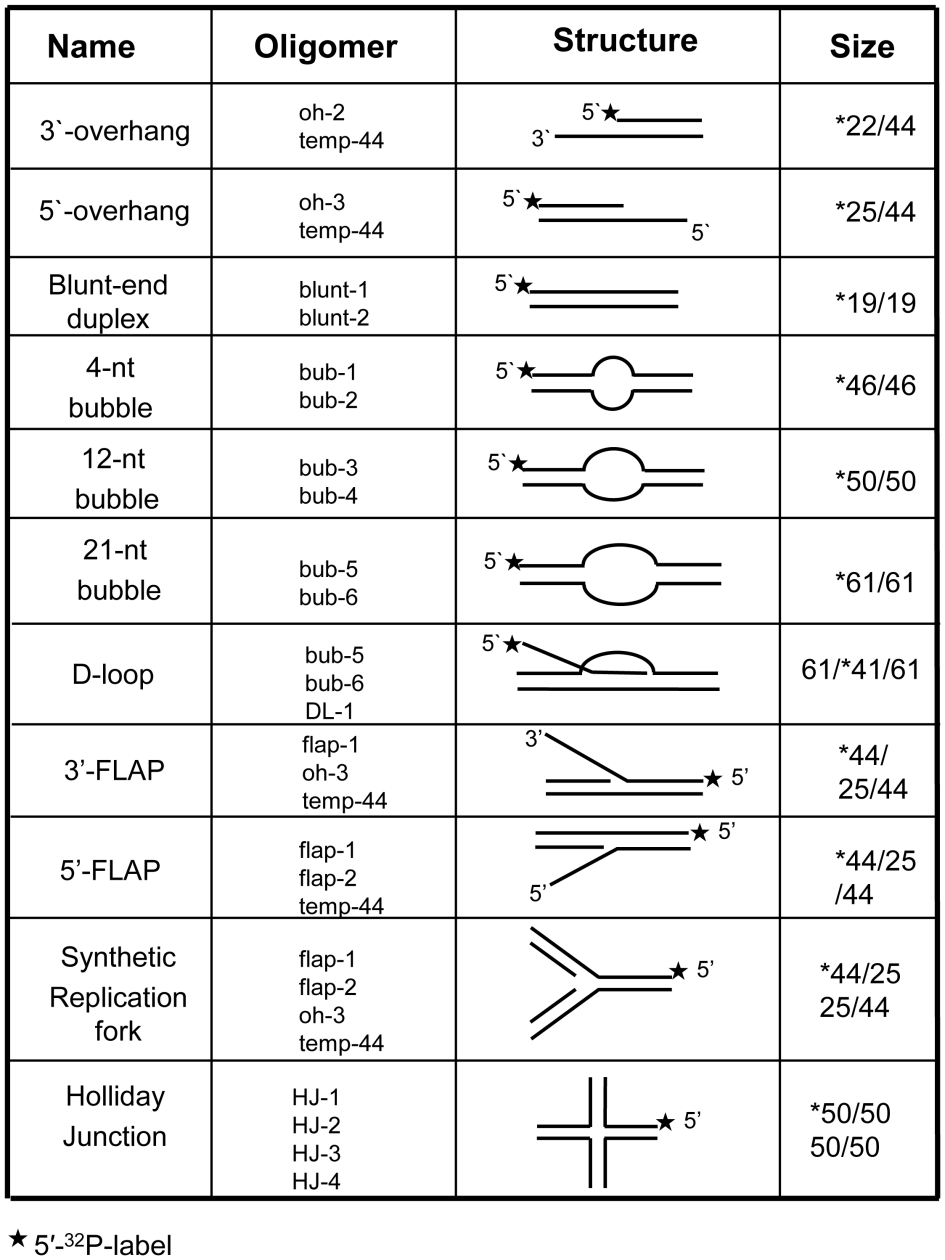
Structures of DNA substrates for helicase assays. The labeled oligonucleotides were annealed to unlabeled complementary strands as described in Materials and Methods.

**Figure 3:**
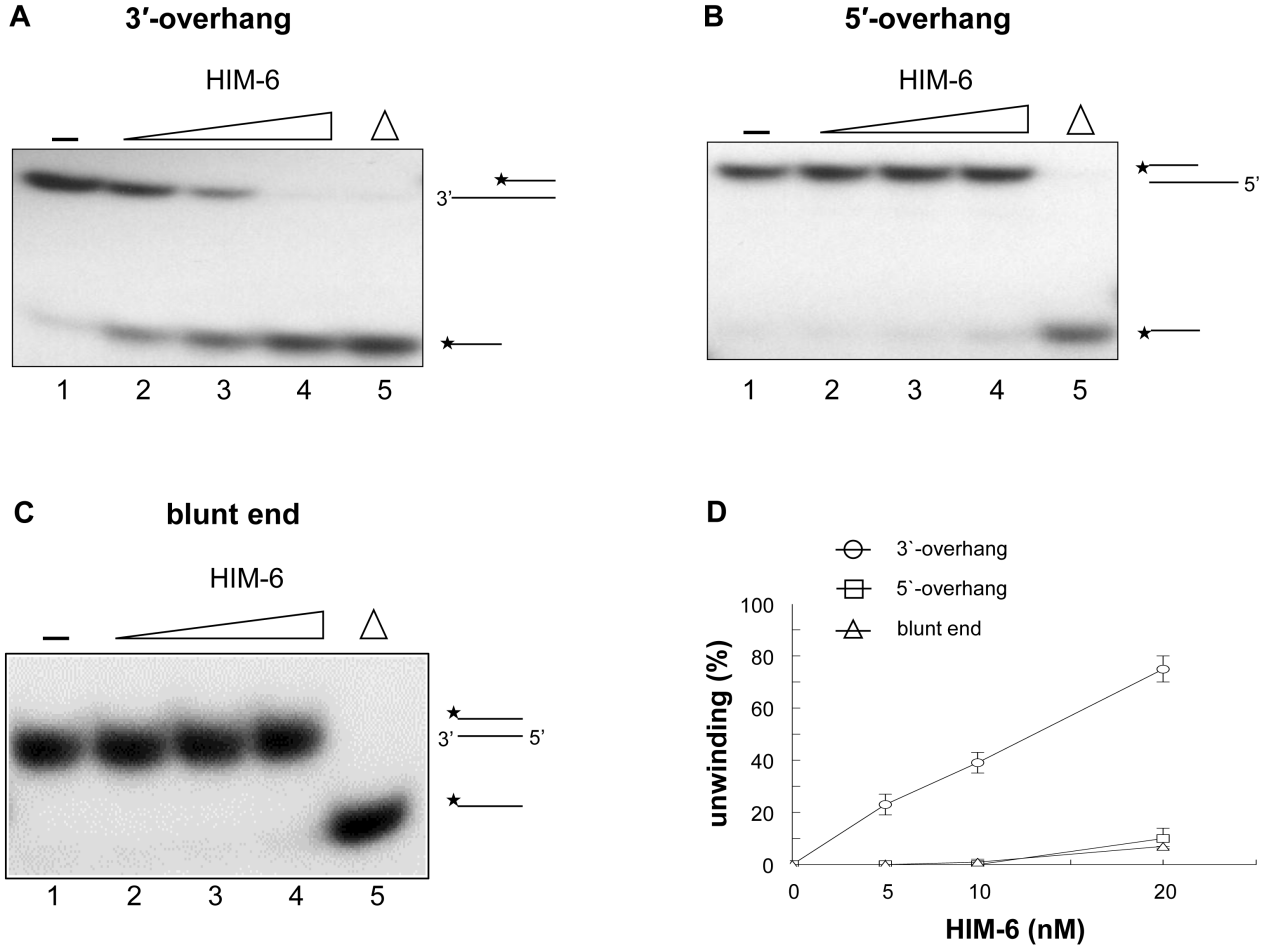
HIM-6 is a 3'-5' DNA helicase. (A) 3'-ssDNA tailed duplex, (B) 5'-ssDNA tailed duplex, and (C) blunt-ended duplex substrates. Helicase reactions were performed with the indicated concentrations of HIM-6 and 1 nM substrates. Lane 1, no enzyme control; lanes 2-4, HIM-6 5 nM, 10 nM, and 20 nM, respectively; lane 5, heat denatured DNA substrate (* indicates the 5'-end labeled with ^3^ ^2^P-label). (D) Quantitation of the data from panels D-F expressed as relative percentages of unwinding. Data are means of at least 3 independent experiments ± SD: (○) 3'-ssDNA tailed duplex; (□) 5'-ssDNA tailed duplex; (▵) blunt-ended duplex.

Because HIM-6 contains conserved helicase motifs from the RecQ helicase family, we first analyzed its ability to unwind 5'-ssDNA overhang, 3'-ssDNA overhang, and blunt-ended duplex substrates. The 3'-overhang substrates and the 5'-overhang substrates are partial duplex oligonucleotides that contain 22 nucleotides of 3'-ssDNA flanked by a 22-base pair dsDNA region or a 19-nucleotide 5'-ssDNA and a 25-base pair dsDNA region, respectively (Figure 2). We found that HIM-6 displaced the 3'-tailed substrate in a concentration-dependent manner (Figure 3A), and 40% strand displacement occurred at a protein concentration of approximately 10 nM (Figure 3D). However, HIM-6 was ineffective at unwinding the DNA substrates with 5'-ssDNA tails or blunt ends (Figures 3B and 3C). These results indicated that HIM-6 bound to the 3'-ssDNA tail, initiated unwinding, and translocated along the bound ssDNA in a 3'-5' direction. Consequently, HIM-6 is a 3'-5' helicase, which is a general characteristic of the RecQ helicase family.

### HIM-6 Helicase Activity on Forked DNA Duplexes

**Figure 4:**
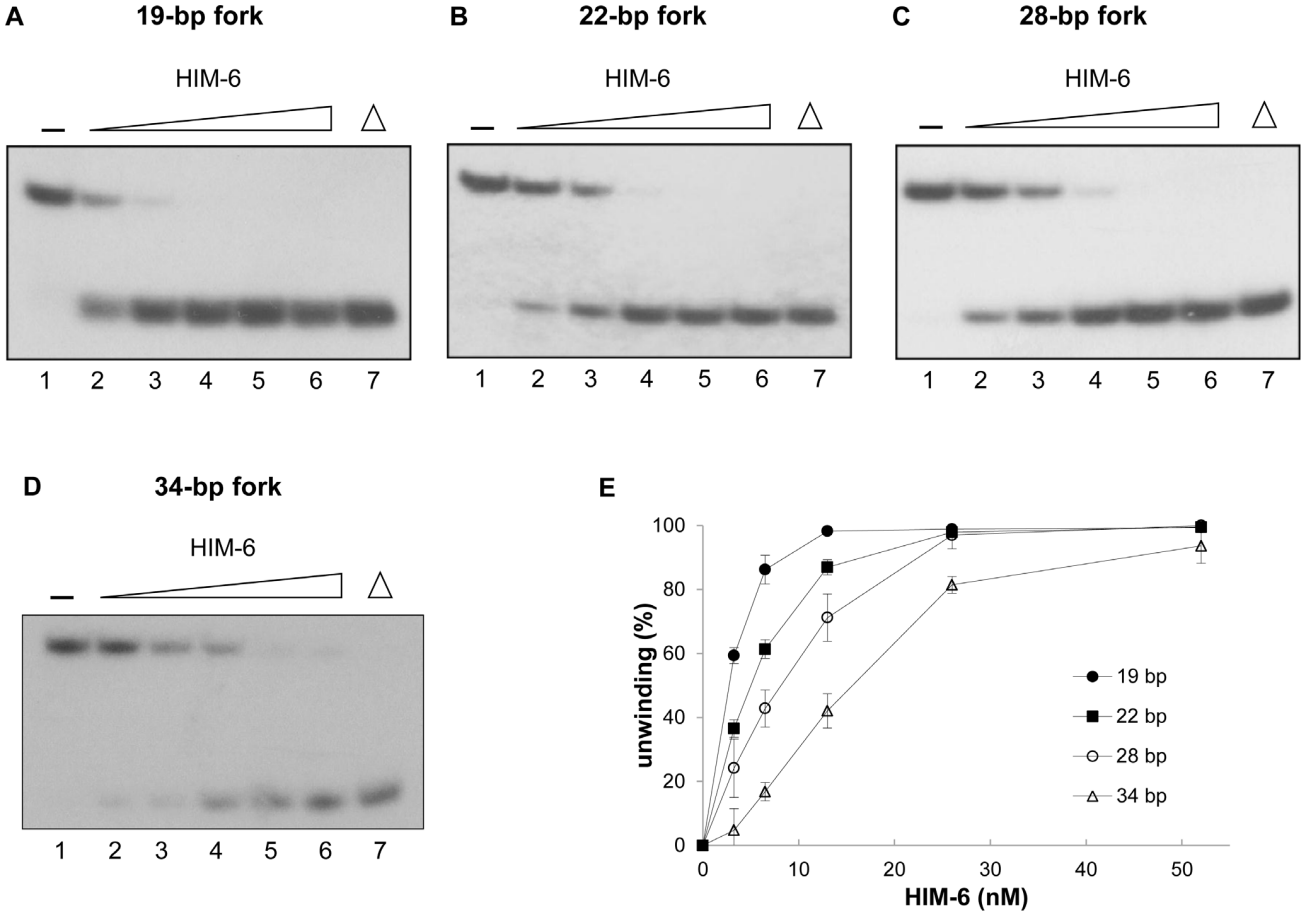
HIM-6 helicase activity on forked DNA substrates with increasing duplex lengths. (A) Helicase reactions with the indicated concentrations of HIM-6 and 1 nM forked duplex substrate lengths (19–34 bp). Lane 1, no enzyme; lanes 2–6, HIM-6 3.25 nM, 6.5 nM, 13 nM, 26 nM, and 52 nM, respectively; lane 7, heat-denatured DNA substrate. (B) Quantification of the data, expressed as means of at least 3 independent experiments ± SD: (•) 19 bp, (▪) 22 bp, (○) 28 bp, (▵) 34 bp.

We next examined whether HIM-6 could unwind forked DNA structures, since other RecQ helicase members have been reported to unwind DNA with fork-structures. We found that HIM-6 catalyzed the unwinding of a 19-bp DNA duplex with 15-nucleotide forks (Figure 4A). At concentrations greater than 6.5 nM, HIM-6 unwound 80–90% of the substrate (Figure 4E). However, when the lengths of the duplex regions were increased (see Figure S1A in File S1 for a detailed description of the substrates), DNA unwinding was significantly reduced using the same protein concentrations (Figures 4B–4D). HIM-6 unwound only 17% of a 34-base pair forked duplex at 6.5 nM (Figure 4E). Interestingly, all substrates were unwound equally well at the highest HIM-6 concentration tested.

Importantly, HIM-6 unwound DNA substrates with fork-structures more efficiently than 3'-ssDNA overhang duplexes (Figures 3C and 4E). The increased rate of unwinding of forked substrates may have occurred because the unpaired ends destabilized the duplex and promoted unwinding. The RecQ helicase, DmBLM, unwound DNA substrates with fork-structures with higher initial rates and to a greater extent than 3'-ssDNA overhang duplexes [14].

### HIM-6 has DNA-dependent ATP hydrolysis activity

**Figure 5:**
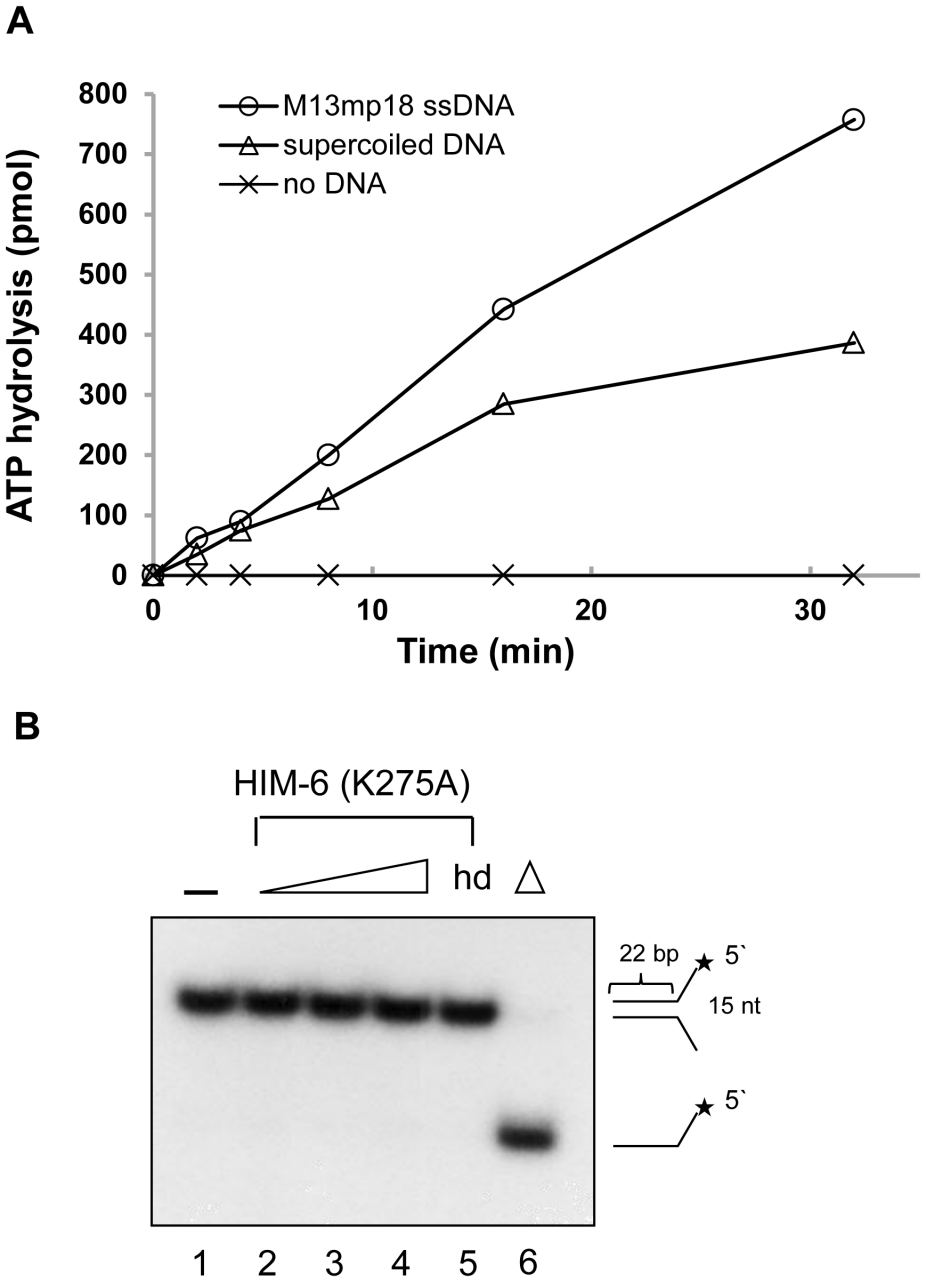
HIM-6 helicase activity is ATPase-dependent. (A) ATPase activity of HIM-6 in the presence of the indicated DNA effectors. Reaction mixtures contained 60 nM HIM-6, 2 mM ATP and 250 ng DNA effector and were incubated at 37°C. (○) circular M13mp18 ssDNA; (□) supercoiled plasmid; (▵) no DNA. (B) Helicase assays for HIM-6 (K275A). Lanes 1, no enzyme; lane 5, heat-denatured protein; lane 6, heat-denatured DNA substrate. Lanes 2–4 contain 5 nM, 10 nM, and 20 nM, respectively.

Because HIM-6 contains the seven commonly conserved helicase motifs, the ATPase activity of HIM-6 was examined in the presence of ssDNA or dsDNA, which are required for ATP binding and hydrolysis in other helicases [3]. The level of ATP hydrolysis was measured using a QuantiChrom ATPase assay. HIM-6 exhibited ATP hydrolysis in the presence of both ssDNA and dsDNA (Figure 5A), although the stimulation was higher with ssDNA. However, ATP hydrolysis was undetectable when DNA was omitted. These results indicated that HIM-6 had DNA-dependent ATPase activity.

We constructed a catalytically inactive HIM-6 mutant. The invariant lysine residue in conserved motif I of the RecQ helicases which is also known as the Walker A motif (TGxGKS, Figure S2A in File S1) is important for ATP hydrolysis and DNA unwinding [22], [23]. The lysine residue (Lys275) of HIM-6 was identified as the corresponding lysine in the Walker A motif according to alignments with BLM (K695) and murine BLM (K703) (Figure S2A in File S1). To produce a catalytically inactive HIM-6 mutant, we replaced Lys275 with an alanine residue, HIM-6 (K275A).

HIM-6 (K275A) was purified identically to the wild-type HIM-6 (data not shown). The HIM-6 (K275A) mutant showed no helicase activity on a forked duplex substrate (Figure 5B) and no ATP hydrolysis in the presence of DNA (Figure S2B in File S1). These data indicated that the conserved lysine 275 was important for DNA unwinding and that the HIM-6 helicase activity was coupled to its DNA-dependent ATPase activity.

### HIM-6 Helicase Activity on 3'-overhang substrates

**Figure 6:**
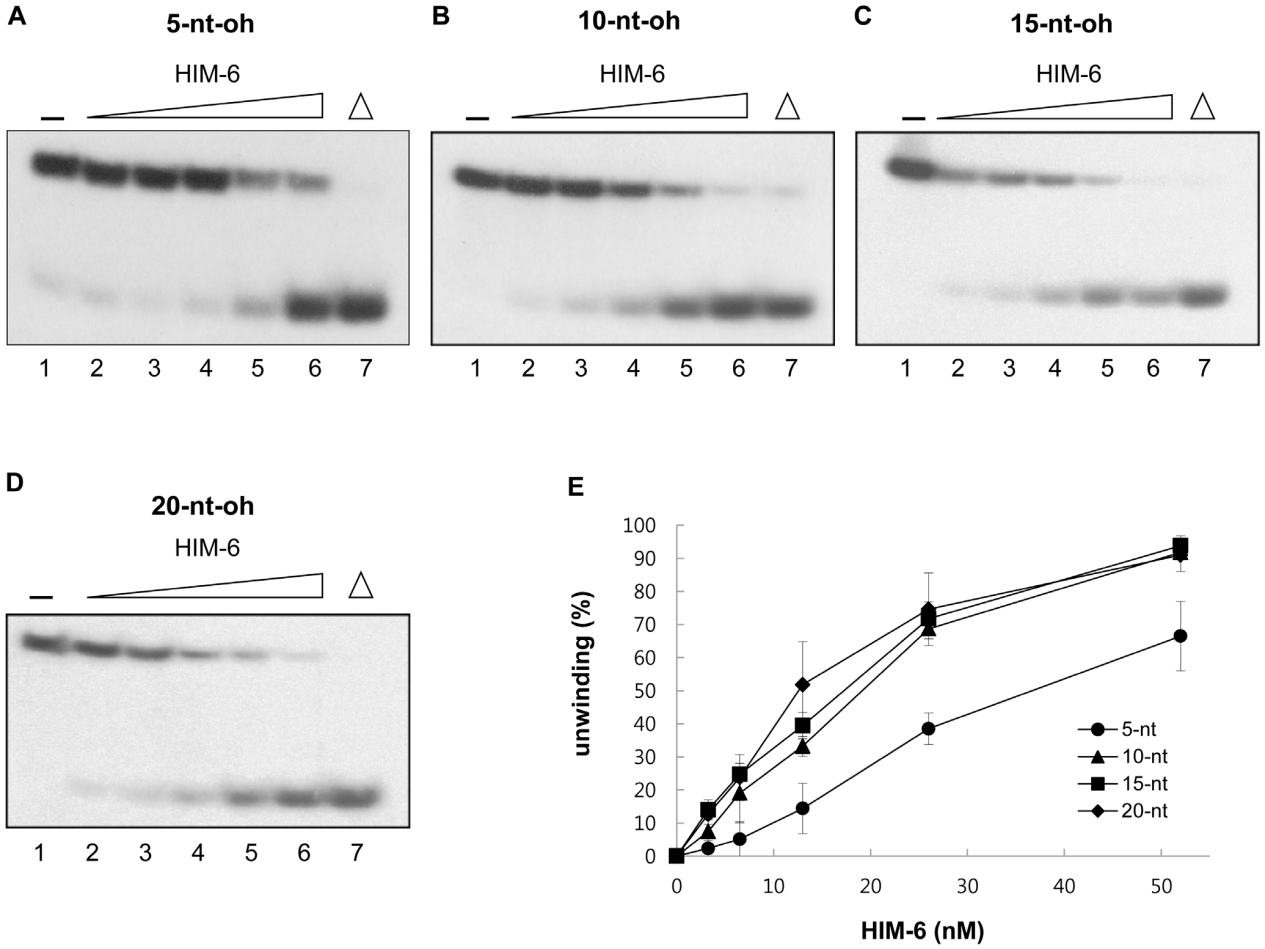
HIM-6 helicase activity on 3′-overhanged DNA substrates with increasing overhang length. (A) Helicase reactions with the indicated concentrations of HIM-6 and 1 nM 3′-ssDNA tailed duplex substrates. Lane 1, no enzyme; lanes 2–6, HIM-6 3.25 nM, 6.5 nM, 13 nM, 26 nM, and 52 nM, respectively; lane 7, heat-denatured DNA substrate. (B) Quantification of the data expressed as means of at least 3 independent experiments ± SD: (•) 5 nucleotides, (▪) 10 nucleotides, (▴) 15 nucleotides, (♦) 20 nucleotides.

Because HIM-6 did not unwind blunt-ended DNA duplexes but unwound a 3'-ssDNA overhang duplex, the effect of the 3'-ssDNA tail length on unwinding was investigated. Substrates were generated with 19-base pair dsDNA portions and 3'-ssDNA overhangs of increasing length (5–20 nucleotides, Figure S1B for a detailed description of the substrates). The substrate containing a 5-nucleotide 3'-overhang was weakly unwound by HIM-6 at protein concentrations of 3.3 nM and 6.5 nM (Figures 6A and 6E). However, a significantly greater proportion of the 10-nucleotide and 15-nucleotide 3'-overhang DNA substrates were unwound by 6.5 nM and 13 nM HIM-6, compared to the 5-nucleotide 3'-overhang duplex (Figures 6B, 6C, and 6E). When the length of the 3'-ssDNA tail was increased to 20 nucleotides, HIM-6-mediated unwinding was even more efficient (Figures 6D and 6E). At the highest HIM-6 protein concentration tested, 52 nM, most of DNA substrates were unwound except for the 5-nucleotide 3'-overhang substrate.

### HIM-6 unwinds bubble structures

**Figure 7:**
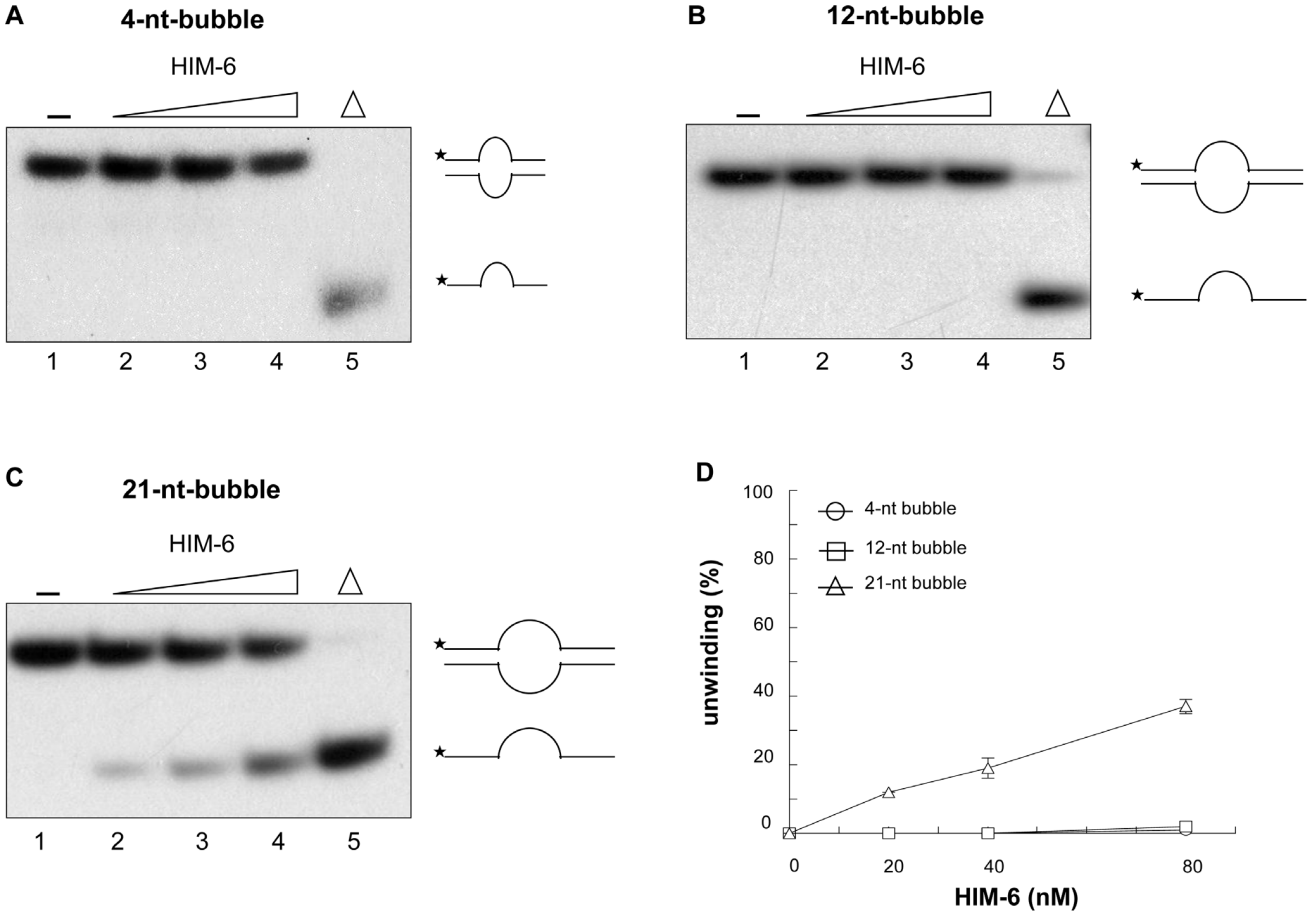
HIM-6 unwinds bubble structures. Helicase reactions with the indicated concentrations of HIM-6 and (A) 1 nM 4-bp bubble, (B) 1 nM 12-bp bubble, and (C) 1 nM 21-bp bubble. Lane 1, no enzyme; lanes 2–4, HIM-6 20 nM, 40 nM, and 80 nM, respectively; lane 5, heat-denatured DNA substrate. The substrates and products are shown schematically on the right. (D) Quantification of the data from panels A–C expressed means of at least 3 independent experiment ± SD: (○) 4-nt bubble, (□) 12-nt bubble, (▵) 21-nt bubble.

The ability of HIM-6 to unwind 3'-overhang and forked duplex substrates indicated that it required a free ssDNA tail to initiate dsDNA unwinding. Therefore, we investigated whether HIM-6 could unwind dsDNA containing an internal ssDNA region. We examined the unwinding of bubble structures that contained single-stranded regions of 4, 12, or 21 nucleotides and dsDNA regions of approximately 19–21-base pairs (Figures 2 and 7). The 4-nucleotide and 12-nucleotide bubble substrates were not unwound (Figures 7A, 7B, and 7D), which suggested that the protein was unable to load at the ssDNA/dsDNA junctions. However, the 21-nucleotide bubble improved the ability of HIM-6 to unwind the bubble substrate (Figures 7C and 7D), suggesting that HIM-6 was able to recognize the ssDNA/dsDNA junction provided by the 21-nucleotide single-stranded DNA region and that HIM-6 did not require a free 3'-ssDNA tail to unwind the bubble structure.

### HIM-6 unwinds Flap substrates and a synthetic replication fork

**Figure 8:**
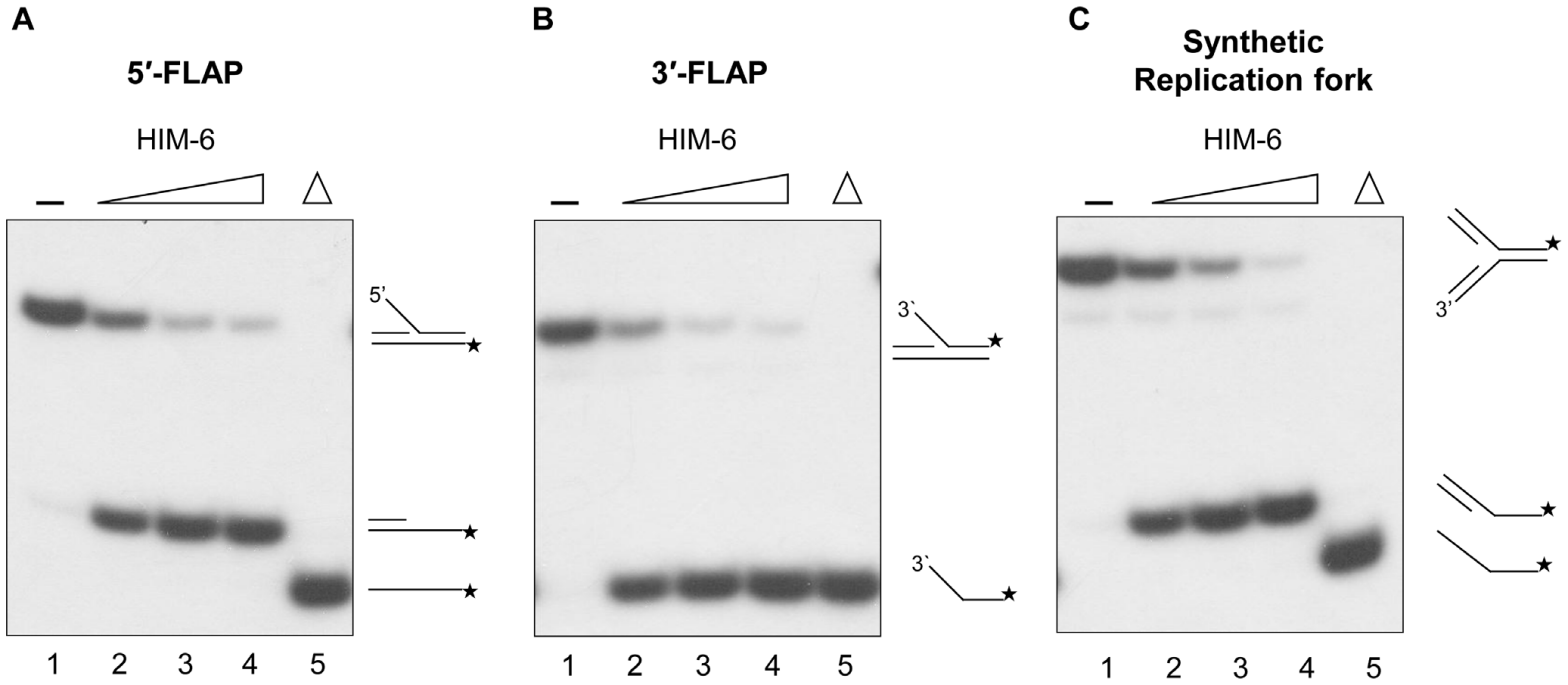
HIM-6 unwinds flap and three-way junction structures. Unwinding of (A) 5′-flap DNA, (B) 3′-flap DNA, and (C) three-way junction substrates. Helicase reactions were performed with the indicated concentrations of HIM-6 and 1 nM substrates. Lane 1, no enzyme; lanes 2–4, HIM-6 2.5 nM, 5 nM, and 10 nM, respectively; lane 5, heat-denatured DNA substrate.

The ability of HIM-6 to unwind forked substrates suggested the possibility that it may be active on another types of branched substrates, including flap structures and three-way junction structures, which have been proposed as intermediates generated during DNA replication or by a stalled replication fork. We tested whether HIM-6 could unwind a 5'-flap substrate, which is a forked substrate with a 5'-ssDNA tail and a 3'-dsDNA tail (Figure 2). A partial DNA duplex was observed as a major reaction product (Figure 8A). This observation indicated that HIM-6 did not require a preexisting free 3'-ssDNA tail adjacent to the duplex region but that it may be able to recognize the type of ssDNA/dsDNA junction.

We also tested whether HIM-6 could unwind a 3'-flap substrate, which is a forked substrate with 3'-ssDNA and 5'-dsDNA tails. All reaction products were 5'-labeled oligonucleotides (Figure 8B), indicating that HIM-6 may bind to the 3'-ssDNA tail and translocate in a 3'-5' direction to produce the unwound product.

We also determined whether HIM-6 could utilize a synthetic replication fork substrate, which mimics a synthetic replication fork with double-stranded leading and lagging strands (Figure 8C). A major unwound product of this substrate was a partial DNA duplex. These data indicated that the HIM-6 helicase recognized the junctions of forked duplexes that lack ssDNA and that unwinding occurred in the direction of the synthetic replication fork thereby separating the two template strands.

### HIM-6 unwinds a displacement loop (D-loop) structure and a HJ structure

**Figure 9:**
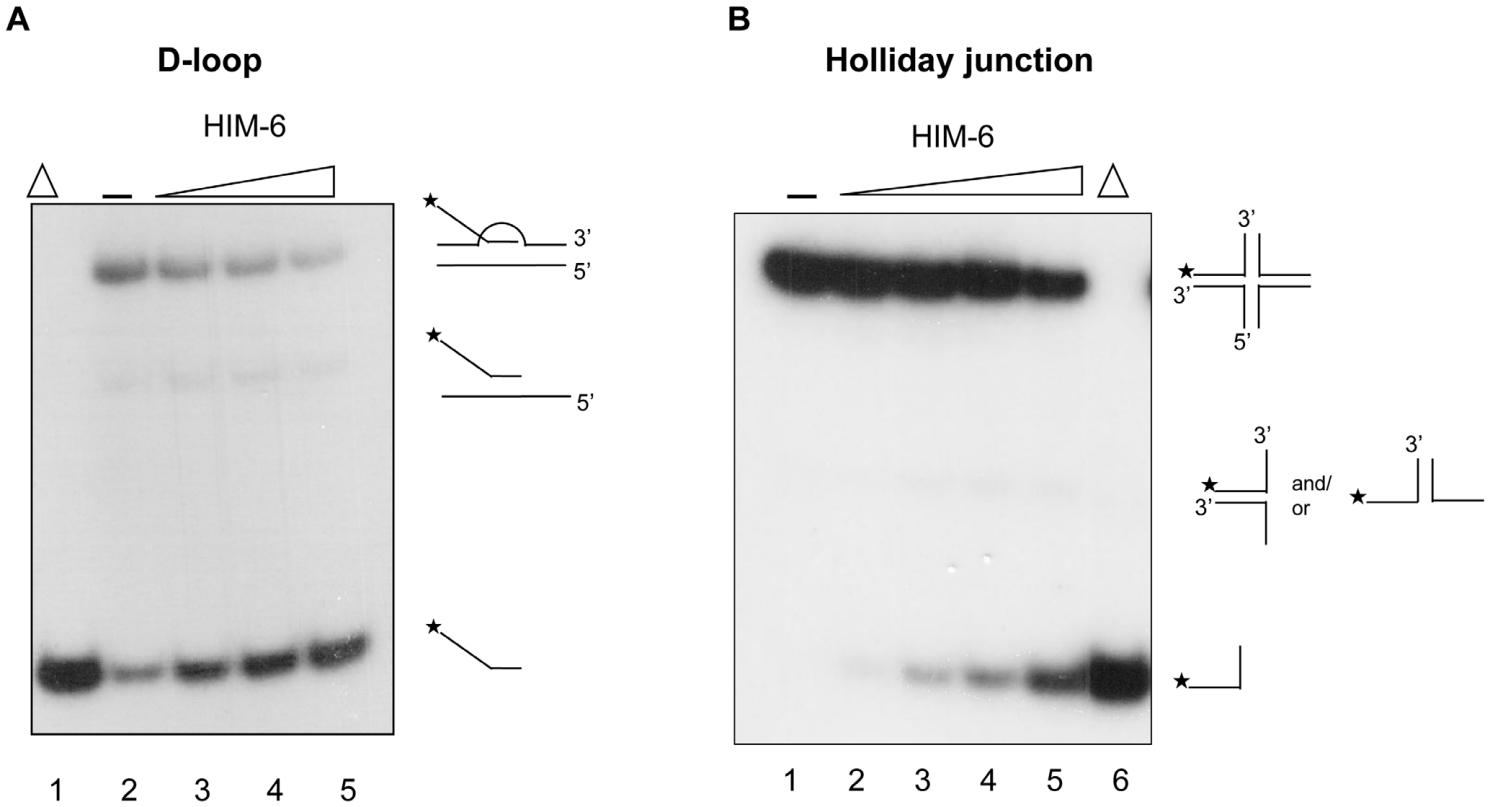
HIM-6 unwinds D-loop and Holliday junction structures. Unwinding of D-loop (A) and HJ (B) structures. Helicase reactions were performed with the indicated concentrations of HIM-6 and 1 nM substrates. Lane 1, no enzyme; lanes 2-5, HIM-6 2.5 nM, 5 nM, 10 nM, and 20 nM, respectively; lane 6, heat-denatured DNA substrate.

D-loops are formed when ssDNA invades duplex DNA at a double-DSB during an early step in homologous recombination (HR) and are maintained after DNA synthesis of the invaded strand. We examined whether HIM-6 could unwind a D-loop structure in which the invaded strand was radiolabeled (Figure 2). The D-loop substrates had some free labeled oligomers because the substrates in this experiment were not gel-purified (lane 2 in Figure 9A). However, the labeled oligomers were greatly dissociated from the tailed D-loop structure and co-migrated with heat-denatured DNA substrate representing the invaded strand (lanes 3-5 in Figure 9A). These data suggested that HIM-6 specifically released the invaded strand from the D-loop structure.

We also analyzed a synthetic X-structure, which is a model of a HJ recombination intermediate in which each arm of the 4-way junction is blunt-ended and is equivalent to a single immobile HJ (Figure 2). HIM-6 was able to unwind the synthetic X-structure to produce a single-stranded oligonucleotide (Figure 9B). Reaction intermediates such as a forked duplex or a structure lacking one strand were not observed at early time points in the reaction (less than 2 minutes, data not shown). These results suggested that HIM-6 recognized the junction and translocated bidrectionally, resulting in a forked duplex. Because HIM-6 could also act upon the fork intermediates, in longer-duration reactions HIM-6 would unwind these structures to produce single-stranded oligonucleotide products.

### Defective recovery from S-phase arrest

**Figure 10:**
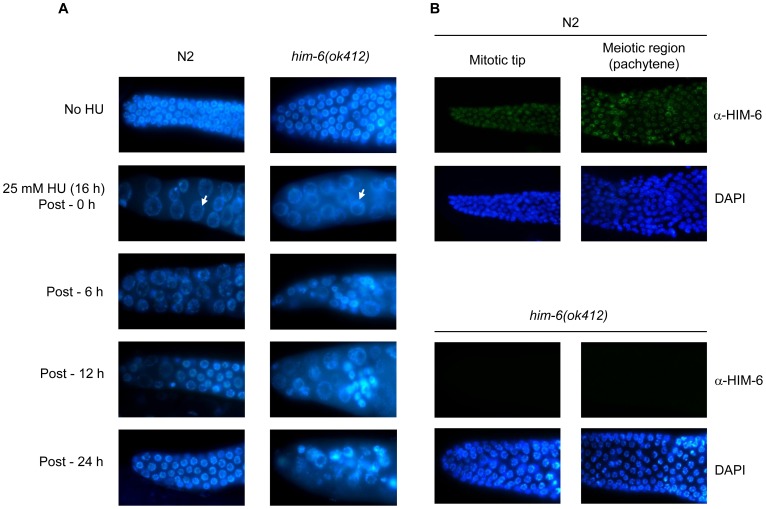
Recovery from cell cycle arrest and Immunolocalization of HIM-6. (A) L4 worms were treated with 25 mM HU for 16 h and the transferred to NGM plates allowing recovery. Dissected gonads from the worms at the indicated times were stained with DAPI. White arrows indicate the enlarged germ cells. (B) Dissected gonads from N2 and *him-6* (*ok412*) were immunostained with HIM-6 antibodies and stained with DAPI.

We investigated the recovery of *him-6* mutants after exposure to HU, a drug that depletes deoxynucleoside triphosphate pools. Worms with *him-6* mutations were grown on plates containing HU, and cell cycle arrest was observed by detecting the size and number of mitotic germ cell nuclei, which indicate cell cycle progression. In this assay, wild-type cells transiently stop dividing in response to HU, and checkpoint-defective cells continue to proliferate [24]. *him-6*(*ok412*) mutants, which contains the deletion allele *him-6*, showed enlarged germ cells, similar to HU-induced cell cycle arrest in wild-type N2 worms. White arrows indicate one example of enlarged germ cells (Figure 10A). However, wild-type N2 worms showed signs of recovery from the cell cycle arrest, whereas reduced recovery was observed in *him-6* mutants. These results indicated that HIM-6 may act in reactivating the cell cycle or repairing the DNA damage induced by HU treatment rather in DNA damage checkpoint.

As recent genetic studies have proposed that HIM-6 may be involved in HJ resolution of meiotic recombination, we examined the localization of HIM-6 in the germline. Dissected gonads from wild-type N2 were immunostained with HIM-6 antibodies. HIM-6 protein was observed in mitotic nuclei at the distal tip region of the germline (mitotic tip) as well as pachytene nuclei (Figure 10B). No staining is observed outside of the gonad (data not shown). However, no signal was detected in these regions in *him-6*(*ok412*), confirming the specificity of the antibody.

## Discussion

We purified recombinant HIM-6 proteins lacking the N-terminal 20 amino acids that do not contain any conserved sequences or motifs. As expected for a RecQ homolog, our data indicated that HIM-6 has DNA-dependent ATPase and 3'-5' DNA helicase activities that can unwind D-loop and HJ structures. These results strongly support a role for HIM-6 in processing recombination intermediates *in vivo*.

BLM homologs function in HR in mitotic and meiotic cells [25], [26]. A D-loop is a strand invasion intermediate of HR-mediated DSB repair, which is generated with a 3′-ssDNA overhang and homologous DNA duplex, and is catalyzed by the Rad51 protein [27]. The invaded DNA is synthesized using the 3′-end as a primer [28]. D-loops can be processed in different ways, with different recombination product outcomes. For example, if the extended nascent strand is displaced from the D-loop and annealed with its original complementary strand, non-crossover (NCO) recombination products are generated by synthesis-dependent strand-annealing (SDSA) mechanisms in mitotic cells [29]. *In vivo* and *in vitro* studies have shown that the yeast helicase Sgs1 displaces D-loops and promotes NCO formation by SDSA [30]–[32]. In addition, the BLM helicase is able to dissociate D-loops made by the human Rad51 protein [7], [33]. These activities suggest that BLM might suppress crossover (CO) recombination products by dismantling D-loops. Thus, the ability of human BLM to unwind a D-loop may be relevant to the hyper-recombination phenotype exhibited by cells from Bloom syndrome patients [34]. Although detailed HR defects have not been studied in *C. elegans him-6* mutants, previous studies showed that *him-6* mutants have enhanced irradiation sensitivity and mitotic chromosomal abnormalities [6]. Our data showing that HIM-6 unwinds D-loops indicates that HIM-6 may disrupt recombination intermediates to promote the mitotic SDSA pathway of HR. Consistent with this model, it has been reported that DmBLM is required for SDSA [12].

D-loops can also be processed to produce dHJs. When a D-loop is stabilized, a dHJ can be formed by capturing the other DSB end. A dHJ can then be processed further to produce a CO or an NCO product. BLM and DmBLM proteins were shown to form complexes with topoisomerase III alpha and disrupt dHJs through branch migration, which is also called dissolution, leading to the separation of the two joined molecules and ultimately the formation of an NCO [10], [15]. In the absence of BLM, resolvase-mediated cellular processes are the dominant pathways for dealing with dHJ structures, which give rise to COs and the exchange of large segments of homologous chromosomes. Indeed, loss of Sgs1 or BLM activity results in increased mitotic COs [35], [36] and may explain the high levels of SCEs in BS cells [37].

The double depletion of *him-6* and *top-3 alpha* genes in *C. elegans* led to a massive increase in DSBs and chromosomal abnormalities in germ cells [6]. In addition, over-expressed TOP-3 alpha and HIM-6 proteins showed specific physical interactions *in vitro* [38]. Based on these genetic and biochemical data, HIM-6 is predicted to participate in the dissolution of dHJs. Our observation that HIM-6 unwound a single HJ may indicate that it is also capable of partially unwinding dHJs. However, to determine whether these *in vitro* HIM-6 activities may promote NCOs at the expense of COs *in vivo*, the TOP-3 alpha and HIM-6 complex needs to be further characterized. In addition, a recent study showed that *him-6* mutations in combination with mutations in the structure-specific nucleases, *mus-8*1 and *slx-1*, produce mitotic defects, including embryonic lethality and larval arrest, and suggest an *in vivo* role for him-6 in processing recombination intermediates [39].

In meiotic recombination, HJs including dHJs can be formed between homologous chromosomes and are initiated by the introduction of programmed DSBs. In yeast, it was been shown that NCOs are formed in an Sgs1-dependent manner [25].

Recently, In *C. elegans*, an important role for HIM-6 in meiotic recombination was reported. Worms with *him-6* and *mus-81* mutations exhibited increased recombination intermediates, suggesting that HIM-6 and MUS-81 may limit early production of recombination intermediates in meiosis [40]. In addition, worms with mutations in *him-6* and *xpf-*1, an endonuclease related to MUS-81, displayed pairs of univalents linked by chromatin bridges representing unresolved meiotic HJs and reduced CO recombination [41]. These data suggest that HIM-6 and XPF-1 may promote HJ resolution and processing of meiotic recombination intermediates leading to CO products. However, exactly how HIM-6 acts with these nucleases remains unclear. Our observations indicated that HIM-6 was able to unwind HJs and D-loops, therefore it is reasonable to speculate that HIM-6 functions to target and/or activate these nucleases at an HJ or a D-loop. Further biochemical studies with HIM-6 and these nucleases will help uncover these roles of HIM-6. Moreover, co-localization with other proteins involved in meiotic recombination, such as XPF-1 and SLX-4, remains to be investigated.

When a replication fork is stalled at a DNA lesion on the leading strand, an HJ can be formed through regression of the nascent leading and lagging strands. This type of HJ can be resolved by repair or bypassed [42]. A recent *in vivo* study showed that human RecQ helicases, WRN and BLM, function in the reactivation of forks after treatment with HU [43]. In addition, BLM localized to repair centers at collapsed replication forks in response to HU [44]. Biochemical studies showed that BLM regresses the stalled replication fork and separates HJ structures [8], [45]. Although *in vivo* data on the reactivation of forks after HU treatment are not currently available in *C. elegans him-6* mutants, our observation, showing recovery of cell cycle arrest after HU treatment, suggests that HIM-6 may operate in repair and recovery from replication fork collapse.

Our studies revealed that HIM-6 dissociated the 5'-flap strand from 5'-flap DNAs. A 5'-flap structure has been suggested as an intermediate in the processing of Okazaki fragments produced by replication or long-patch base excision repair [46]. Biochemical studies showed that human WRN and BLM interact with flap endonuclease-1 (FEN-1) to stimulate flap endonuclease activity [47]–[49]. The *C. elegans* homolog of human FEN-1, CRN-1, can also cleave 5'-flap structured DNA [50]. Thus, we predict that HIM-6 coordinates with CRN-1 and assists in processing flap structured DNA.

We also determined that HIM-6 was capable of unwinding double-stranded 3-way junction DNA that mimics replication forks to produce partial-duplex products (probably 5'- and 3'-overhang partial duplexes). With the polarity of HIM-6, it may translocate along the leading strand to separate the two template strands ahead of the fork structure. CeWRN-1 and WRN have also been shown to unwind 3-way junctions in that way [22], [51]. Although biochemical studies of human BLM and DmBLM at 3-way junctions have not been reported, human BLM was recently shown to function in normal replication fork progression *in vivo* [43]. Our data showing that HIM-6 unwinds the 3-way junction toward the replication fork suggests that HIM-6 may participate in replication fork progression.

Taken together, our results revealed HIM-6 as a DNA helicase with roles in processing recombination intermediates. Thus, it will be interesting to uncover *in vitro* activities of HIM-6 with other interacting proteins to address how HIM-6 is involved in HR in *C. elegans*.

## Supporting Information

File S1 **Figures S1 & S2.** Figures S1A and S1B. Structures of DNA Substrates for Helicase Assays. The labeled oligonucleotides were annealed to unlabeled complementary strands as described in Materials and Methods. Figure S2A. A Sequence alignment between human, murine, and *C. elegans* BLM holomogs. A domain containing Walker A-type (GXGGKS) is conserved. Nucleotide sequences (aaa) for lysine residue (275) of HIM-6 was mutated to nucleotide sequences (gcg) for alanine residue. Figure S2B. ATPase activity of HIM-6 (K275A) mutant. Reaction mixtures contained HIM-6 (K275A), 2 mM ATP, and 250 ng/μl DNA effector and were incubated at 37°C for 30 min. The amount of inorganic phosphate (Pi) released by ATP hydrolysis was determined as described in the Experimental procedures. X, no DNA; ○, circular M13mp18 ssDNA.
(PDF)
