# Supplementary material for: Characterization of the Caenorhabditis elegans HIM-6/BLM Helicase: Unwinding Recombination Intermediates
Source: PLoS One. 2014 Jul 18;9(7):e102402. doi: 10.1371/journal.pone.0102402 (PMC4103807; doi:10.1371/journal.pone.0102402)
Supplement: File S1 — Figures S1 & S2. Figures S1A and S1B. Structures of DNA Substrates for Helicase Assays. The labeled oligonucleotides were annealed to unlabeled complementary strands as described in Materials and Methods. Figure S2A. A Sequence alignment between human, murine, and C. elegans BLM holomogs. A domain containing Walker A-type (GXGGKS) is conserved. Nucleotide sequences (aaa) for lysine residue (275) of HIM-6 was mutated to nucleotide sequences (gcg) for alanine residue. Figure S2B. ATPase activity of HIM-6 (K275A) mutant. Reaction mixtures contained HIM-6 (K275A), 2 mM ATP, and 250 ng/μl DNA effector and were incubated at 37°C for 30 min. The amount of inorganic phosphate (Pi) released by ATP hydrolysis was determined as described in the Experimental procedures. X, no DNA; ○, circular M13mp18 ssDNA. (PDF) [file pone.0102402.s001.pdf]

Table S1: The sequences of oligonucleotides used for Helicase assays

Oligonucleotide	Length (nt)	Sequence (5'-3')
blunt-1	19	ACCACCCTTCGAACCACAC
blunt-2	19	GTGTGGTTCGAAGGGTGGT
bub-1	46	CGAGCTCGGTACCCGGGGATCCTCTAGAGTCGACCTGCAGGCATGC
bub-2	46	GCATGCCTGCAGGTCGACTCTAGAGGATGGGGGGTACCGAGCTCG
bub-3	50	CGGCTCAACGTGGGCAAAGCCAATGCGATCGGCCAGAATTCGGCAGCGTC
bub-4	50	GACGCTGCCGAATTCTGGCTTGCTCGGACATCTTTGCCCACGTTGACCCG
bub-5	61	GACGCTGCCGAATTCTACCAGTGCCTTGCTAGGACATCTTTGCCCACCTGCAGGTTCACCC
bub-6	61	GGGTGAACCTGCAGGTGGGCGGCTGCTCATCGTAGGTTAGTTGGTAGAATTCGGCAGCGTC
DL-1	41	TTTTTTTTTTTTTTTTTTTTCCGACGAGTAGCATCCAATCA
fork-01	34	CGTAACGCGTAACGCGTAATTTTTTTTTTTTTTT
fork-02	34	TTTTTTTTTTTTTTTTTACGCGTTACGCGTTACG
fork-03	37	TTTTTTTTTTTTTTTTTAGGGTTAGGGCATGCACTAC
fork-04	37	GTAGTGCATGCCCTAACCCTAATTTTTTTTTTTTTTT
fork-07	43	CGCGTAACGCGTAACGCGTAACGCGTAATTTTTTTTTTTTTTT
fork-08	43	TTTTTTTTTTTTTTTTTACGCGTTACGCGTTACGCGTTACGCG
fork-09	46	GTGCGCGTAACGCGTAACGCGTAACGCGTAATTTTTTTTTTTTTTT
fork-10	46	TTTTTTTTTTTTTTTTTACGCGTTACGCGTTACGCGTTACGCGCAC
fork-11	49	TTTTTTTTTTTTTTTTTACGCGTTACGCGTTACGCGTTACGCGCACTAC
fork-12	49	GTAGTGCGCGTAACGCGTAACGCGTAACGCGTAATTTTTTTTTTTTTTT
flap-1	44	TTTTTTTTTTTTTTTTTTTTTTCAATTAAGAATTCGGCAGCGTC
flap-2	25	TTGAAAAAAAAAAAAAAAAAAAAAA
HJ-1	50	GACGCTGCCGAATTCTGGCTTGCTAGGACATCTTTGCCCACGTTGACCCG
HJ-2	50	CGGGTCAACGTGGGCAAAGATGTCCTAGCAATGTAATCGTCTATGACGTC
HJ-3	50	GACGTCATAGACGATTACATTGCTAGGACATGCTGTCTAGAGACTATCGC
HJ-4	50	GCGATAGTCTCTAGACAGCATGTCCTAGCAAGCCAGAATTCGGCAGCGTC
oh-1	19	TTAAGAATTCGGCAGCGTC
oh-2	25	ACGACCTTAAGAATTCGGCAGCGTC
oh-3	25	CGCCAGGGTTTAGATGTTCACGACC
oh-4	24	GACGCTGCCGAATTCTTAAGGTCG
oh-5	29	GACGCTGCCGAATTCTTAAGGTCGTGAAC
oh-6	34	GACGCTGCCGAATTCTTAAGGTCGTGAACATCTA
oh-7	39	GACGCTGCCGAATTCTTAAGGTCGTGAACATCTAAACCC
temp-44	44	GACGCTGCCGAATTCTTAAGGTCGTGAACATCTAAACCCTGGCG

Fork duplex length (bp)	Oligomer	Structure	Size
19-bp-fork	fork-1<br>fork-2	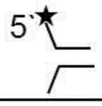	*34/34
22-bp-fork	fork-3<br>fork-4	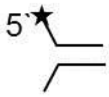	*37/37
28-bp-fork	fork-7<br>fork-8	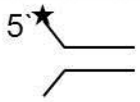	*43/43
34-bp-fork	fork-11<br>fork-12	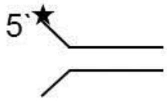	*49/49

Figure S1A. Structures of forked DNA Substrates for Helicase Assays

3’-overhang	Oligomer	Structure	Size
5-nt-oh	oh-1<br>oh-4	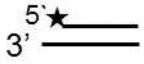	*19/24
10-nt-oh	oh-1<br>oh-5	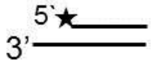	*19/29
15-nt-oh	oh-1<br>oh-6	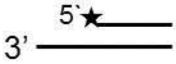	*19/34
20-nt-oh	oh-1<br>oh-7	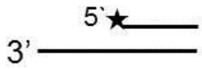	*19/39

Figure S1B. Structures of 3’-overhang DNA Substrates for Helicase Assays

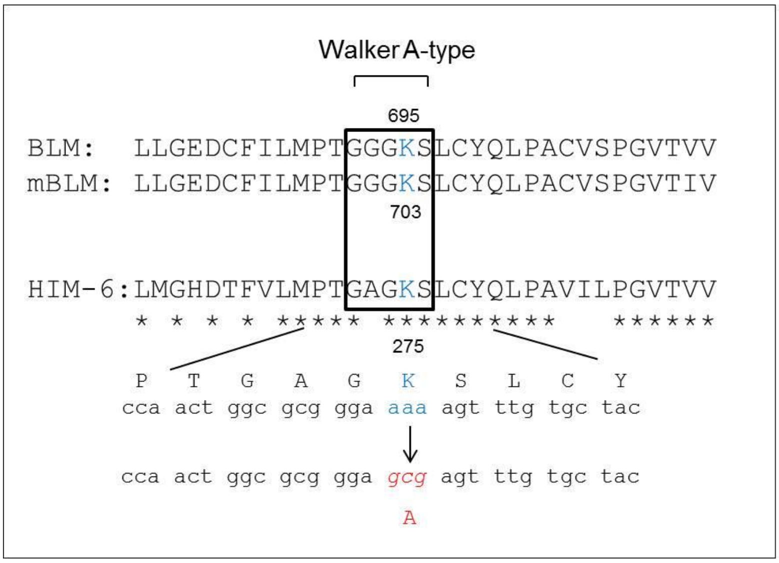

Figure S2A. A Sequence alignment between human, murine, and *C. elegans* BLM holomogs. .

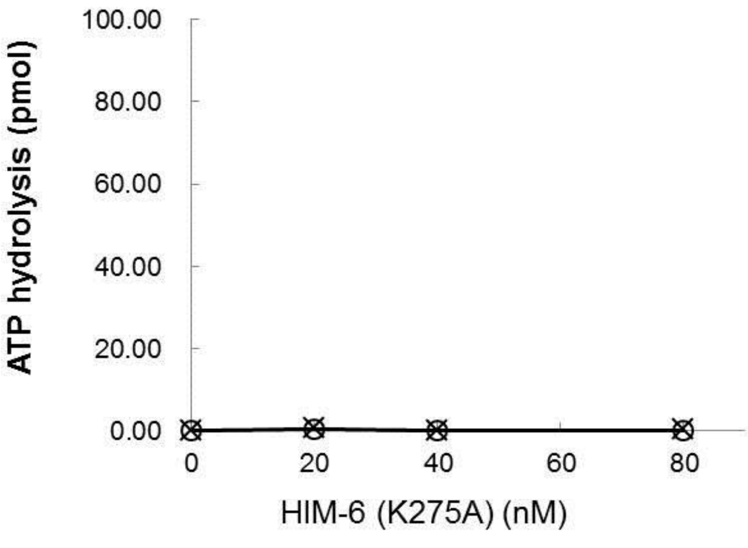

Figure S2B. ATPase activity of HIM-6 (K275A) mutant
